# Rubber tail illusion is weakened in Ca^2+^-dependent activator protein for secretion 2 (*Caps*2)-knockout mice

**DOI:** 10.1038/s41598-019-43996-9

**Published:** 2019-05-17

**Authors:** Makoto Wada, Masakazu Ide, Takeshi Atsumi, Yoshitake Sano, Yo Shinoda, Teiichi Furuichi, Kenji Kansaku

**Affiliations:** 1 Developmental Disorders Section, Department of Rehabilitation for Brain Functions, Research Institute of National Rehabilitation Center for Persons with Disabilities, Tokorozawa, 359-8555 Japan; 2 Faculty of Informatics, Shizuoka University, Hamamatsu, 432-8011 Japan; 3 Department of Medical Physiology, Faculty of Medicine, Kyorin University, Tokyo, Mitaka 181-8611 Japan; 4 Department of Applied Biological Science, Tokyo University of Science, Noda, 278-8510 Japan; 5 Department of Environmental Health, School of Pharmacy, Tokyo University of Pharmacy and Life Sciences, Hachioji, 192-0392 Japan; 6 Systems Neuroscience Section, Department of Rehabilitation for Brain Functions, Research Institute of National Rehabilitation Center for Persons with Disabilities, Tokorozawa, 359-8555 Japan; 7 Department of Physiology and Biological Information, Dokkyo Medical University School of Medicine, Mibu, 321-0293 Japan; 8 Brain Science Inspired Life Support Research Center, University of Electro-Communications, Chofu, 182-8585 Japan

**Keywords:** Cognitive neuroscience, Sensorimotor processing, Psychology

## Abstract

Body ownership is a fundamental aspect of self-consciousness. Illusion of body ownership is caused by updating body representation through multisensory integration. Synchronous visuotactile stimulation of a hand and rubber hand leads to illusory changes in body ownership in humans, but this is impaired in individuals with autism spectrum disorder (ASD). We previously reported that mice also exhibit body ownership illusion. With synchronous stroking of a tail and rubber tail, mice responded as if their own tails were being touched when the rubber tails were grasped (‘rubber tail illusion’). However, it remains unknown whether deficits in illusion of body ownership occur in mouse models of autism. Here, we examined whether the ‘rubber tail illusion’ occurred in *Ca*^2+^*-dependent activator protein for secretion* 2*-*knockout (*Caps2*-KO) mice, which exhibit autistic-like phenotypes. During the synchronous stroking, response rates were significantly lower in *Caps2*-KO mice than in wild-type mice. There were no significant differences between the response rates of wild-type and *Caps2*-KO mice during the asynchronous stroking. The ‘rubber tail illusion’ was weak in *Caps2*-KO mice, suggesting that *Caps2*-KO mice experienced weaker visuotactile integration during the task. The rubber tail task will be a useful tool in mouse models of autism to evaluate atypical sensory processing.

## Introduction

Body ownership is fundamental to self-consciousness and is crucial for environmental interactions in daily life. We sometimes feel a sense of body ownership for objects outside the body. For example, when a rubber hand and participant’s hand are synchronously stroked by two brushes, participants feel as if the rubber hand becomes their own hand (the rubber hand illusion)^1–4^. In such situations, integration of simultaneous visual and tactile stimuli is thought to change the perception of body ownership. Thus, changes in body ownership are thought to be caused by multisensory integration. Individuals with autism spectrum disorder (ASD) have difficulty experiencing aspects of the rubber hand illusion^5,6^. Paton *et al*.^6^ reported that individuals with ASD showed reduced sensitivity to visuotactile-proprioceptive discrepancy, but more precise proprioception during the illusion. Cascio *et al*.^5^ reported that children with ASD exhibited difficulty experiencing the illusion initially; however, after more than 5 minutes of stroking, they experienced the effects of the illusion. Thus, the occurrence of the rubber hand illusion was delayed. In addition, we recently found that individuals with high autistic traits in neurotypicals experience a weaker illusory body ownership of the rubber hand during the illusion, and a lower concentration of salivary oxytocin is associated with this weaker illusory body ownership^7^. As indicated by several different tasks^8,9^, greater reliance on proprioception is thought to inhibit such body ownership illusions. Therefore, we speculated that individuals with high autistic traits would tend to form weaker associations between vision and touch, unlike that between touch and proprioception.

Meanwhile, previous studies have suggested that non-human animals may also experience body ownership illusions. Shokur *et al*.^10^ suggested that synchronous visual and tactile stimuli presented to an avatar’s and monkey’s hand caused activation of neurons in the somatosensory cortex by ‘touching’ the avatar’s hand. Thus, macaque monkeys may experience the rubber hand illusion of the avatar’s hand in such situations^10^. By analogy to the rubber hand illusion, we previously examined whether mice experience similar illusions. In our experiment, we delivered synchronous stroking to an artificial tail (rubber tail) and the real tail of a mouse. We found that mice responded (e.g., orienting or retracting the head) as if their own tails were being touched when the rubber tails were grasped (a ‘rubber tail illusion’ in mice)^11^. Thus, it is possible that mouse models of autism may show dysfunctions of body representation similar to human individuals with ASD^5,6^. The use of mouse models enables us to elucidate the neural and genetic bases of deficits in body ownership. Previously, conventional tests for such mice have focused on social communication and repetitive behaviours. For example, social abilities are often examined with a social interaction test or social recognition test using a three-chambered apparatus. Capabilities for auditory communication are typically evaluated by focusing on ultrasonic pup calls, and repetitive behaviours are tested by measuring grooming behaviors^12–14^. To date, no studies have evaluated such aspects of ASD in mouse models of autism. Here, we evaluated impairment of body ownership in a mouse model of autism using the novel ‘rubber tail illusion’ in mice.

Several human neuroimaging studies have suggested that multisensory integration in the posterior parietal cortex is critical for the rubber hand illusion, while the ventral premotor cortex and insular cortex are also involved^15,16^. In the macaque monkey, bimodal neurons that are activated by either visual or tactile stimuli were found in the posterior parietal cortex, and the visual receptive fields of such neurons moved to the tip of a tool when the monkey used a rake-like tool to obtain a food reward^17^. The same investigators also reported that expression of brain-derived neurotrophic factor (BDNF) in the posterior parietal cortex plays an important role during plastic changes in body representations, since BDNF expression was found increased after macaque monkeys were trained in tool use^18^. Moreover, a human electroencephalography study reported that BDNF concentration in peripheral blood correlated with oscillatory components in the high frequency (gamma) band in the posterior parietal cortex during the body ownership illusion^19^. These studies indicated that BDNF expression levels are linked to plastic changes in body representations caused by multisensory integration. It would therefore be intriguing to examine the ‘rubber tail illusion’ in mouse models of autism that have impaired BDNF-related function in the cerebral cortex.

Previous studies have indicated that the BDNF activity in the posterior parietal cortex is important for body ownership illusions. Thus, the mouse in models of autism demonstrating dysfunctional BDNF secretion would serve as good targets for initial evaluation of the ‘rubber tail illusion’. The Ca^2+^-dependent activator protein for secretion (CAPS) protein was first identified as a novel 145 kilodalton (kDa) brain cytosolic protein, which reconstitutes Ca^2+^-regulated secretion in permeable neuroendocrine cells^20^. Compared with that of the CAPS1 protein, expression of the CAPS2 protein in the brain is limited to several regions and cell types (e.g., interneurons in the cerebral cortex, neurons in the dentate gyrus, and granular cells in the cerebellar cortex)^21^. Subsequent studies indicated that the CAPS2 protein enhanced the release of BDNF. Moreover, its deficiency in mice impaired the development of inhibitory interneurons, leading to cellular and electrophysiological dysfunction that resembles that observed in human developmental disorders^22^. Consistent with these findings, *Caps2*- knockout (KO) mice exhibit autistic-like phenotypes, and variants of the *CAPS2* gene have been found in some autistic families^23,24^.

Therefore, among the various mouse models of autism, the *Caps2*-KO mouse, which exhibit impairments in BDNF release, represents a suitable first target for evaluating body ownership using the rubber tail task.

## Results

**Figure 1:**
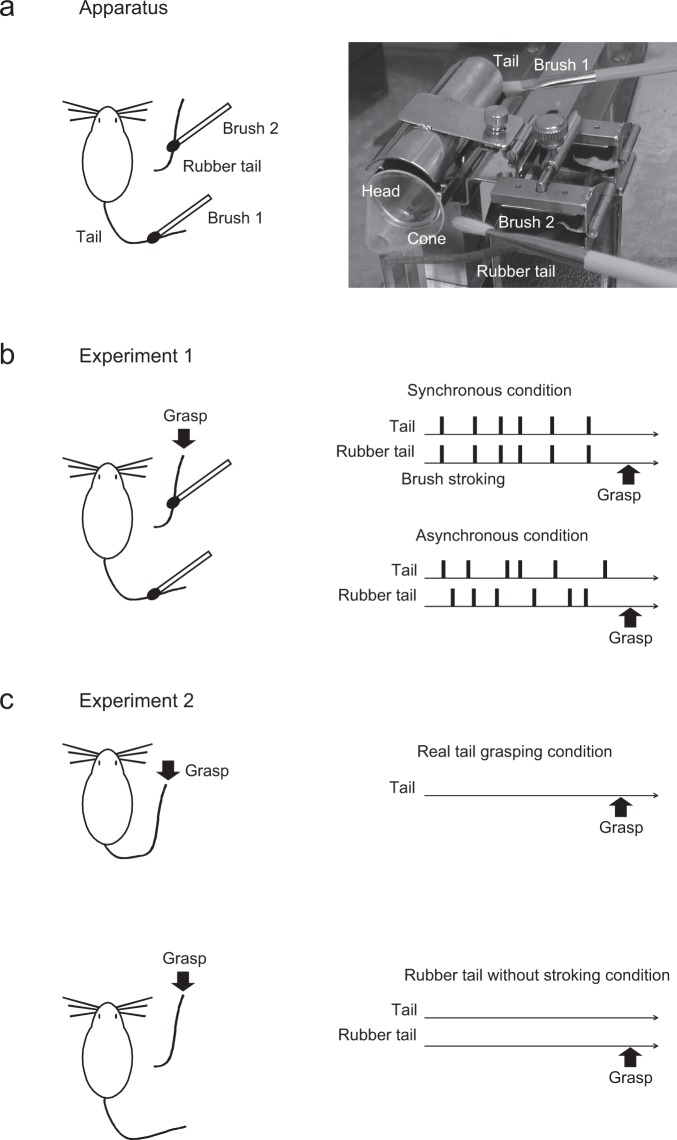
Apparatus and task schedules. (**a**) Apparatus: a tail and rubber tail received brush stroking in a stainless-steel tube as shown in the right panel. One side of the tube was open, and the other side of the tube was connected to a transparent plastic cone. A rubber tail was placed either to the right or left of the cone. (**b**) Experiment 1: Synchronous vs Asynchronous conditions. In the Synchronous condition, both *Caps2*-KO and Wild-type mice received synchronous stroking to real tails and rubber tails using two small brushes for 1 minute (0.5–2 Hz). In contrast, they received asynchronous stroking in the Asynchronous condition for 1 minute (0.5–2 Hz). The apparatus was almost identical to those in our previous study^11^. (**c**) Experiment 2: Real tail grasping condition and rubber tail without stroking condition. In the real tail grasping condition, responses to real tail grasping without any stroking were examined. In the rubber tail without stroking condition, responses to rubber tail grasping without any brush stroking were examined.

In this study, we examined whether the ‘rubber tail illusion’ occurred in *Caps2*-KO mice and wild-type (Wild-type) mice (Fig. 1).

### ‘Rubber tail illusion’ was absent in Caps2-KO mice

Mice were trained and tested in a stainless-steel tube designed for the rubber tail task (Fig. 1a). Mice received initial training to stay in the tube. In the rubber tail task (Fig. 1b), mice were subjected to daily tests, for 10 minutes under two conditions using two small brushes. The Synchronous condition involved synchronous stroking of real and rubber tails, whereas the Asynchronous condition involved asynchronous stroking of both tails. After the tails were stroked for approximately 1 minute, an experimenter who was blinded to experimental conditions firmly grasped the rubber tail, and the response of the mouse was evaluated.

First, the responses from the 15 sessions (days) were averaged for each condition. After testing the normality (Shapiro–Wilk test) in both Synchronous (Wild-type: *W* = 0.93, *p* = 0.35; *Caps2*-KO: *W* = 0.93, *p* = 0.32) and Asynchronous conditions (Wild-type: *W* = 0.94, *p* = 0.50; *Caps2*-KO: *W* = 0.95, *p* = 0.54), a two-way analysis of variance (ANOVA) with genotype (*Caps2*-KO or Wild-type) and condition (Synchronous or Asynchronous) as the factors, and using the averaged response rates, were conducted. Although a significant main effect of genotype (*F* = 8.96, *p* = 0.0065, *partial η*^*2*^ = 0.28) suggested that the Wild-type mice showed higher response rates pertaining to head movements, neither the main effect of condition (*F* = 0.65, *p* = 0.43, *partial η*^*2*^ = 0.028) nor the interaction between genotype and condition (*F* = 2.47, *p* = 0.13, *partial η*^*2*^ = 0.097) was significant. These observations suggested the existence of large individual differences in the time courses of the response rates, and therefore, were further evaluated.

**Figure 2:**
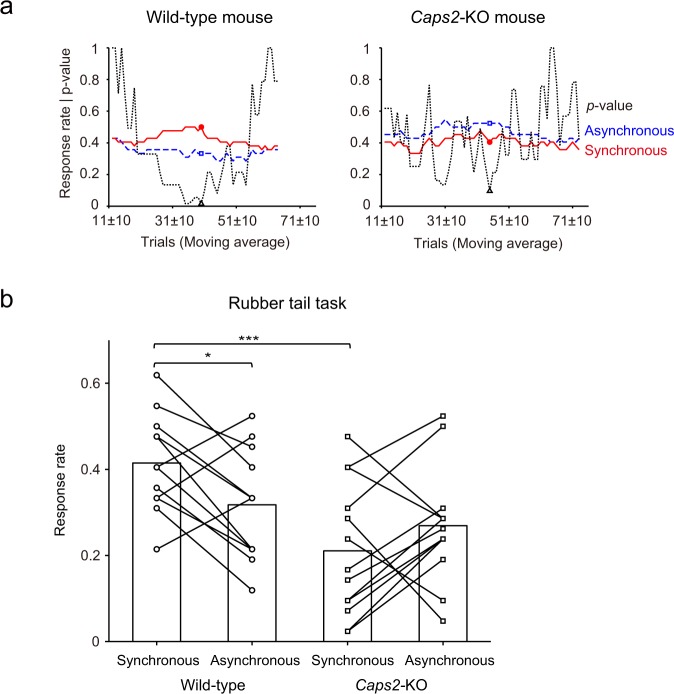
Response rates for the rubber tail task in *Caps2*-KO and Wild-type mice (Experiment 1). (**a**) Representative data of moving averages of response rates in the Synchronous condition (Synchronous; red solid lines) and Asynchronous condition (Asynchronous; blue dashed lines) during the rubber tail task. Note that values in ‘11 ± 10’ indicate mean response rates from trials 1 to 21 (a moving average of 21 trials) in each condition. In addition, black dotted lines indicate p-values between responses in the Synchronous and Asynchronous conditions for each point. (**b**) Response rates in the rubber tail task in each group. As described above, we calculated data points whereby the differences between response rates in the Synchronous and Asynchronous conditions were maximal in each mouse, and a response rate in each condition for each mouse was extracted. Bar graphs indicate averaged values from 12 Wild-type mice and 13 *Caps2*-KO mice. Each marker indicates the averaged value of responses for each mouse in each condition. As described in the Results section, the response rates were significantly high in the Synchronous condition in the Wild-type mice (*p < 0.05, ***p < 0.001), while there was no significant difference between the response rates of Synchronous and Asynchronous conditions in *Caps2*-KO mice.

In order to determine the maximum difference in response rates between the Synchronous and Asynchronous conditions, moving averages before and after 10 trials (every 21 trials) were calculated. We extracted the data point at which the difference between the response rates of the Synchronous and Asynchronous conditions was maximal in each mouse (minimum p-values between them) (Supplementary Table 1). Figure 2a shows changes in the moving averages of each condition and p-values of their differences in a representative Wild-type and *Caps2*-KO mouse, respectively. In the Wild-type mouse, the response rate in the Synchronous condition was significantly higher than that in the Asynchronous condition (*t*_20_ = 2.65, *p* = 0.016, one-sample t-test, 95% *confidence interval* = [0.071, 0.60], *d* = 0.58; Fig. 2a). Eight of the 12 Wild-type mice showed significantly higher response rates in the Synchronous condition (Supplementary Table 1). In contrast, only one of the 13 *Caps*2-KO mice showed this tendency, whereas six *Caps*2-KO mice showed significantly higher response rates in the Asynchronous condition (Supplementary Table 1). In *Caps*2-KO mice, the rubber tail illusion was rarely observed. Data points were averaged, revealing that mean response rates for Wild-type mice in the Synchronous and Asynchronous conditions were 0.41 ± 0.066 and 0.32 ± 0.074 (mean ± standard error), respectively. In contrast, the mean response rates for *Caps*2*-*KO mice in the Synchronous and Asynchronous conditions were 0.21 ± 0.085 and 0.27 ± 0.073, respectively (Fig. 2b).

The estimated response rates were validated using a bootstrapping method. The data from the Wild-type and *Caps*2-KO mice were randomly permutated for each condition and the moving averages were calculated similarly. We observed larger differences in the rates between the Synchronous and Asynchronous conditions with the permutated data (Synchronous–Asynchronous) than that obtained from real situation (positive values in average) of the Wild-type mice for 7 times of 1000 repetitions (*p* = 0.007). Contrarily, we observed more negative values in the permutated data than in the real situation (negative values in average) of the *Caps*2-KO mice for 171 times of 1000 repetitions (*p* = 0.17). Additionally, we observed larger differences than in the real situation (for Wild-type mice) only for 15 times out of 1000 repetitions (*p* = 0.015), as compared to the differences (Synchronous– Asynchronous) between the Wild-type and *Caps*2-KO mice. Thus, the differences observed for Wild-type mice were validated, and were not coincidental.

After testing for normality (Shapiro–Wilk test) in both Synchronous (Wild-type: *W* = 0.98, *p* = 0.99, *Caps2*-KO: *W* = 0.92, *p* = 0.27) and Asynchronous conditions (Wild-type: *W* = 0.94, *p* = 0.52, *Caps2*-KO: *W* = 0.90, *p* = 0.15), we conducted a two-way ANOVA with genotype (*Caps2*-KO or Wild-type) and condition (Synchronous or Asynchronous) as factors using these rates. A significant main effect of genotype (*F* = 8.40, *p* = 0.0081, *partial η*^*2*^ = 0.27) suggested that Wild-type mice showed higher response rates of head movements, while the main effect of condition was not significant (*F* = 0.39, *p* = 0.54, *partial η*^*2*^ = 0.017). A significant interaction between genotype and condition (*F* = 6.36, *p* = 0.019, *partial η*^*2*^ = 0.22) suggested a significant difference in response rates between genotypes among the conditions.

In the Synchronous condition, a significant simple main effect of genotype (*F* = 14.0, *p* = 0.001, *partial η*^*2*^ = 0.38) suggested that the response rates of *Caps2*-KO mice in the Synchronous condition were significantly smaller than those in Wild-type mice. In contrast, response rates in the Asynchronous condition were not significantly different between groups (*F* = 0.85, *p* = 0.37, *partial η*^*2*^ = 0.035).

In Wild-type mice, a significant simple main effect of condition (*F* = 4.95, *p* = 0.036, *partial η*^*2*^ = 0.18) suggested that the response rates of Wild-type mice in the Synchronous condition were significantly larger than those in the Asynchronous condition, consistent with our previous study^11^. In contrast, response rates were not significantly different between conditions in *Caps2*-KO mice (*F* = 1.80, *p* = 0.19, *partial η*^*2*^ = 0.073).

### Response rates of real tail grasping were not significantly different between in Caps2-KO and Wild-type mice

**Figure 3:**
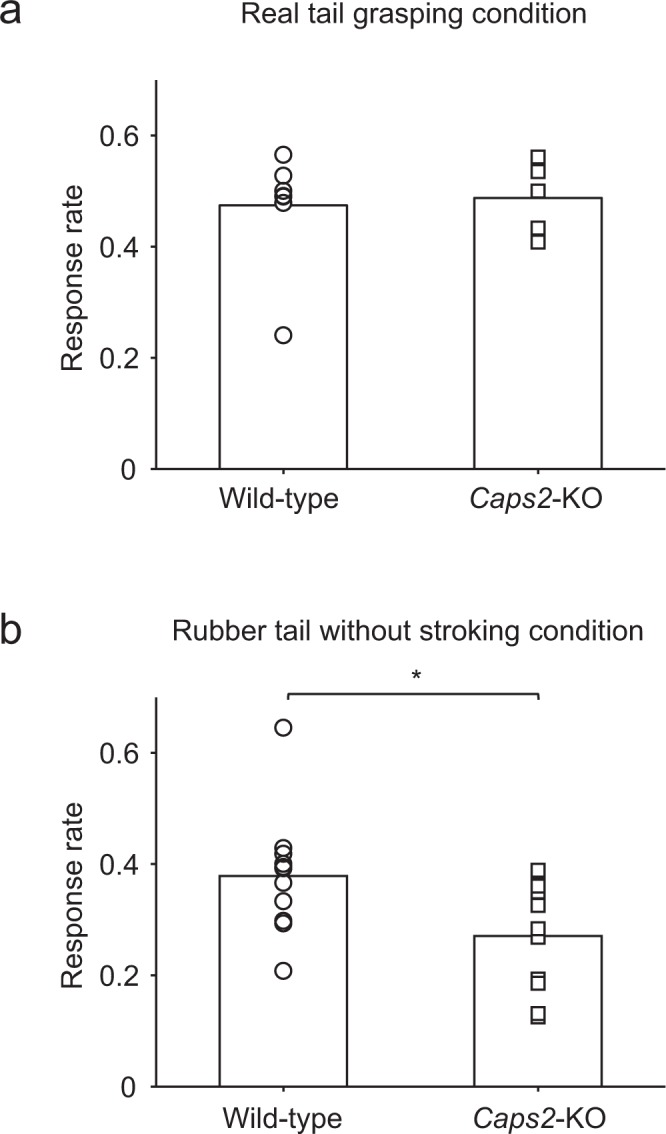
Response rate of real tail grasping condition and rubber tail without stroking condition (Experiment 2). (**a**) Real tail grasping condition. Each bar indicates the mean response rate of six sessions per mouse for each group (Wild-type mice: n = 8, *Caps2*-KO mice: n = 5). No significant difference between groups was detected. (**b**) Rubber tail without stroking condition. Each bar indicates the mean response rate of five sessions per mouse in each group (Wild-type mice: n = 12, *Caps2*-KO mice: n = 11). As described in the Results section, the mean response rate was significantly smaller in *Caps2*-KO mice than in Wild-type mice (*p < 0.05). Each marker indicates the averaged value of responses for each mouse in each condition.

Responses to real tail grasping were examined in a subset of mice in the real tail grasping condition without any brush stroking (Fig. 1c). In this condition and in the absence of the rubber tail, the real tail was placed on the right side of each mouse and fixed with surgical tape. During the 10-min test, the real tail was grasped at intervals of approximately 1 minute. When the real tail was grasped in both *Caps2*-KO (n = 5) and Wild-type mice (n = 8), similar responses (Fig. 3a, *Caps2*-KO mice: 0.47 ± 0.035, Wild-type mice: 0.48 ± 0.029, respectively) to the rubber tail illusion were observed. Owing to the non-normal distribution of the Wild-type mice dataset on Shapiro–Wilk test, a non-parametric method was used in this experiment for further analysis (Wild-type: *W* = 0.68, *p* = 0.0013, *Caps2*-KO: *W* = 0.93, *p* = 0.62). We found that there was no significant difference between them (*W* = 21, *p* = 0.94, Wilcoxon rank sum test, 95% *confidence interval* = [−0.082, 0.071], *d* = 0.16).

We also examined responses when the rubber tail was grasped without synchronous and asynchronous stroking (Fig. 1c). During the 10-min test, the rubber tail was grasped at intervals of approximately 1 minute. In this case, the normality of each data was not rejected (Wild-type mice: *W* = 0.88, *p* = 0.088, *Caps2*-KO: *W* = 0.90, *p* = 0.18, Shapiro–Wilk test). The response rate (0.27 ± 0.029, n = 11; Fig. 3b) of *Caps2*-KO mice was significantly lower than that of Wild-type mice (0.38 ± 0.030, n = 12; *t*_21_ = −2.56, *p* = 0.018, two-sample t-test, 95% *confidence interval* = [−0.20, −0.020], *d* = 1.07).

## Discussion

In the present study, the response rates of *Caps2*-KO mice in the Synchronous condition were lower than those of Wild-type mice. Further, response rates were not significantly different between Synchronous and Asynchronous conditions in *Caps2*-KO mice. These results suggest that the rubber tail illusion may be weak or impaired in *Caps2*-KO mice. In humans, body ownership illusions including the rubber hand illusion are thought to be caused by multisensory integration of simultaneous visual and tactile stimuli^1,4,25^. We speculated that visuotactile integration would be atypical in *Caps2*-KO mice, although this mouse model exhibits some aspects of normal sensory function^24^. We observed that dysfunction of body ownership in ASD^5,6^ were partially simulated in this mouse model of ASD.

We also examined reactivity to actual and rubber tails without any stroking in experiment 2 in order to evaluate reactivity to tactile or visual stimulation. We observed that response rates were significantly low in the *Caps2*-KO mice when the rubber tail was grasped without stroking. However, general loss of vision in *Caps2*-KO mice is unlikely, since a previous study showed normal sensory function in *Caps2*-KO mice^24^. Instead, in the open field test with a novel object, total distance travelled and the percentage of time spent in the centre of the field were significantly decreased in *Caps2*-KO mice^22^. Thus, ‘novel’ situations (i.e., staying in the tube with the presence of the rubber tail) may inhibit responses in *Caps2*-KO mice. In contrast, in experiment 1, there was minimal difference between the Synchronous and Asynchronous conditions in the *Caps2*-KO mice. The present results imply that deletion of the *Caps2* gene in *Caps2*-KO mice may attenuate the rubber hand illusion.

Previous studies have suggested that the CAPS2 protein is involved in BDNF release and the development of inhibitory interneuron networks^22^. We speculate that this impairment may be related to the absence of the rubber tail illusion in *Caps2*-KO mice. Changing body representations is likely underpinned by plastic changes in neural networks. In such situations, BDNF would be important for remodelling of local circuits, including inhibitory interneurons^18^. In a recent human electroencephalography study, the abundance of BDNF was correlated with oscillatory components in the high frequency (gamma) band in the left parietal cortex during tests related to body ownership illusions^19^. The posterior parietal cortex is thought to contribute to multisensory integration during the rubber hand illusion in humans^15,16^ and is important for spatiotemporal updating of body representation^26^. Dysfunction in BDNF release in these regions may inhibit plasticity in body schema during visuotactile integration. If this is indeed the case, individuals with ASD who have variants in the *CAPS2* gene^24,27^ may exhibit dysfunctions of body ownership relative to those who do not carry such variants.

The role of the *CAPS2* gene during development should also be considered to understand how *CAPS2* gene mutations may disrupt the development of body ownership in individuals with ASD. Neural circuits are thought to be formed in early life stages and are closely related to the pathogenesis of ASD. As BDNF is a neurotrophic factor, dysfunction in BDNF secretion in *Caps2*-KO mice may affect the brain during specific developmental stages. We speculate that impaired development of interneuron networks^22^ in relevant brain regions may be related to deficits in body ownership illusions. In addition to *Caps2*-KO mice, many mouse models of autism are known to have dysfunctions of inhibitory neurons^28^. Excitatory-inhibitory imbalances in neural networks are emerging as plausible factors contributing to the risk for ASD^29–31^, as this process may affect plastic changes in body representation after specific multisensory integration. Testing other strains of mice in autism models in future studies will help to elucidate the molecular underpinnings of body ownership.

Previous studies in mice revealed that CAPS2 protein expression in the brain is confined to specific cell types in several regions (e.g., neurons in the dentate gyrus, interneurons in the cerebral cortex, and granular cells in the cerebellar cortex)^21^. Meanwhile, human imaging studies have suggested that the posterior parietal cortex, ventral premotor cortex, and insular cortex are activated during body ownership illusions including the rubber hand illusion^15,16,32–34^. In addition to dysfunction in BDNF secretion, dysfunctions of inhibitory interneurons in these cerebral areas may also be related to the absence of the rubber tail illusion in *Caps2*-KO mice. Future functional neuroanatomical studies using immediate early genes (e.g., c-fos, arc)^35,36^ or physiological measurements may elucidate the neural basis of body ownership dysfunction in ASD. Future double labelling studies will enable elucidation of cell properties and screening of potential task-related regions.

There may have been potential bias in testing due to manual grasping of the rubber tail by the experimenter. We considered the possibility of bias by checking the response rates of the Asynchronous condition in both groups. As described in the Methods, the experimenter who grasped the rubber tail was blinded to experimental conditions. Thus, if the experimenter had grasped the rubber tail softly only in the *Caps2*-KO mice, response rates in the Asynchronous condition should theoretically also be decreased. The results indicated that the response rates in the Asynchronous condition in *Caps2*-KO mice were not significantly different from those in the Wild-type mice, thereby minimising the possibility of bias. Nevertheless, the possibility of potential bias in all behavioural testing cannot be completely ruled out. Complete blinding and automation of tests in the future will help to eliminate bias.

In this study, we observed that rubber tail illusions were attenuated in *Caps2*-KO mice. We speculate that dysfunction in BDNF release in the brain may impair plastic changes in body schema during visuotactile integration. In addition to the well-known deficits in social communication in ASD patients, the neural basis of various dysfunctions in body ownership and higher cognitive functions may be elucidated in the future by using the rubber tail task with neurophysiological and histological methods.

## Methods

### Animals

*Caps2*-KO mice (n = 13) and Wild-type (n = 12) male mice were used in this study. Four Wild-type mice were littermates of the *Caps2*-KO mice that were bred at Tokyo University of Science. The remaining Wild-type mice were C57BL/6NCrj (Charles River Laboratories Japan Inc., Yokohama, Japan). Details regarding the production and phenotypes of the *Caps2*-KO mice have been described previously^23,24^. Mice were moved from these institutes to our facility by special animal carriers (Sankyo Labo Service Corporation, Inc., Tokyo, Japan; Oriental Yeast Co., Ltd., Tokyo, Japan). Behavioural testing started when mice were 5-weeks-old (15–20 grams). Prior to performing tasks, mice were handled by experimenters for at least 3 days. Mice received daily testing sessions (one session per day) on weekdays and were provided *ad libitum* access to water and food. The mice were housed in individually ventilated plastic cages (Lab Products, Inc., Seaford, DE, USA) with bedding made of paper. Mice were housed in groups of 2–4 littermates in a room kept at 23 °C and 30–50% humidity with a 12 h light/dark cycle. All behavioural experiments were conducted during the light cycle. Prior to behavioural testing, the mice had no history of drug administration or surgery. After concluding experimentation, mice were sacrificed by overdose of anaesthetics (isoflurane or pentobarbital). The experiments were approved by the institutional committee for animal experimentation (Research Institute of the National Rehabilitation Center for Persons with Disabilities), and all experiments were performed in accordance with relevant regulations and guidelines of Ministry of Health, Labour and Welfare of Japan.

### Apparatus

Mice were trained and tested in a stainless-steel tube (30 mm in diameter) that was designed for the rubber tail task (O′Hara, Tokyo, Japan). One side of the tube was open, and the other side of the tube was connected to a transparent plastic cone (Fig. 1a). A rubber tail was placed either to the right or left of the cone. Prior to behavioural testing, mice were trained to stay in a small tube with their heads stationary for over 10 minutes. The apparatus for behavioural testing has been described previously^11^.

### Experiment 1: Rubber tail task (Synchronous and Asynchronous conditions)

In this experiment, in order to evaluate deficits of body ownership in a mouse model of autism, we examined whether the rubber tail illusion was diminished in *Caps2*-KO mice. After initial training to stay in the tube, in the rubber tail task (Fig. 1b), mice (13 *Caps2*-KO and 12 Wild-type mice) were subjected to 10-min daily tests under two conditions, each with two small brushes: 1) synchronous stroking of the real tail and a rubber tail (Synchronous condition), and 2) asynchronous stroking of both tails (Asynchronous condition). The stroking was manually delivered using brushes (0.5–2 Hz), and a stroking lasted approximately 0.5–1 second. After the tails were stroked for approximately 1 minute, an experimenter who was blinded to experimental conditions firmly grasped the rubber tail. The experimenter was instructed to grasp the tail in the same manner at every trial. We conducted the rubber tail task for 15 days. The order of conditions was counterbalanced between sessions.

Two experimenters conducted the experiments. One experimenter controlled the timing of stroking and grasping by observing the second hand of a stopwatch, and the timing of grasping was communicated by using a hand gesture to the other experimenter for grasping who sat facing away from the mouse during stroking. If head of mice moved away from the cone, the experimenter for the stroking kept stroking the rubber tail after the returning to the cone, and continued to stroke the tails for another 1 minute. Thus, the total number of trials in each mouse was less than 150 (Supplementary Table 1).

### Experiment 2: Real tail grasping condition and rubber tail without stroking condition

In this experiment, we examined reactivity to the actual tails and rubber tails without any stroking to evaluate the reactivity of mice to tactile or visual stimulation. In the real tail grasping condition (Fig. 1c), the response to real tail grasping was examined in a subset of mice (five *Caps2*-KO mice and eight Wild-type mice) before or after the rubber tail task. Without any brush stroking, the tail of each mouse was placed on the right side of the mouse without the presence of the rubber tail and fixed with surgical tape. During testing which lasted 10 minutes, the real tail was grasped at intervals of approximately 1 minute. We conducted this test for 6 days and calculated the mean response rate for each mouse.

In the rubber tail without stroking condition (Fig. 1b), responses of the mice (11 *Caps2*-KO mice and 12 Wild-type mice) when the rubber tail was grasped, without any synchronous or asynchronous stroking, was examined. During testing which lasted 10 minutes, the rubber tail was grasped at intervals of approximately 1 minute. The test was conducted before the rubber tail task. We then calculated the mean response rate for each mouse using 5 days of response data.

### Analysis

Head movements of the mice were recorded using a digital video camera (GZ-RX500-B, JVC KENWOOD Corporation, Yokohama, Japan) and evaluated by technical staff blinded to experimental conditions. According to a previous study^11^, if the head promptly turned toward the rubber tail or retracted into the tube, this was considered to be a full response and was given a full score (1.0). If the response was relatively small or delayed (~1 s), this was considered to be an intermediate response and was given a partial score (0.5). Moreover, trials in which mice did not respond were given a score of zero (0). Averaged responses were calculated for each condition.

First, we averaged these responses for all sessions, as in our previous study. However, we found gradual changes in response rates with time (Fig. 2a) and also found larger individual differences in time sequences. We assumed that these changes reflected plasticity during multisensory integration and large differences in the response rates between the conditions indicated acquisition of the illusion. Thus, we decided to use the point where this plastic change was maximal for further analysis. In addition, the delayed occurrence of proprioceptive drift has been described in a study examining human participants with ASD^5^. If a similar phenomenon occurs in *Caps2*-KO mice, simple averaging among several sessions is not a suitable method for comparison between *Caps2*-KO and Wild-type mice.

For further analysis, moving averages before and after 10 trials (every 21 trials) of the response were calculated in the rubber tail task. We then extracted the data point at which the difference between the response rates of the Synchronous and Asynchronous conditions was maximized for each mouse (minimum p-values between conditions, one-sample t-test). Among response rates of all subjects, we conducted a test of normality (Shapiro–Wilk test). After testing the normality of the data, we performed ANOVA with Genotype (*Caps2*-KO, Wild-type) and Condition (Synchronous, Asynchronous) factors using these rates in the rubber tail task (Experiment 1). Data analysis was conducted using Matlab (MathWorks, Natick, MA, USA), R (3.3.2 GUI 1.68), G*Power (version 3.1.9.2) and js-STAR (version 9.0.0j).

## Supplementary information

Supplementary Table 1

Dataset

## Data Availability

The datasets used and/or analysed during the current study are available on a supplementary data sheet.
