# Supplementary material for: Rubber tail illusion is weakened in Ca2+-dependent activator protein for secretion 2 (Caps2)-knockout mice
Source: Sci Rep. 2019 May 17;9:7552. doi: 10.1038/s41598-019-43996-9 (PMC6525187; doi:10.1038/s41598-019-43996-9)
Supplement: Supplementary file 1 — Supplementary Table 1 [file 41598_2019_43996_MOESM1_ESM.docx]

**Rubber tail illusion is weakened in Ca^2+^-dependent activator protein for secretion 2 (*Caps2*)-knockout mice**

Makoto Wada^1, 2*^, Masakazu Ide^1^, Takeshi Atsumi^1^, Yoshitake Sano^3^, Yo Shinoda^3, 4^, Teiichi Furuichi^3^, Kenji Kansaku^5, 6, 7^

**Supplementary Table 1. Individual data of each mice (Experiment 1).**

Total number: total numbers of the trials in the Synchronous and Asynchronous conditions, Data point: the point at which the difference between the response rates of the moving averages (data point ± 10 trials) in the Synchronous and Asynchronous conditions was maximum (minimum p-values between them), Synchronous: response rate of the Synchronous condition in the point, Asynchronous: response rate of the Asynchronous condition in the point, p-value: p-values from one-sample t-test of the response rates between the conditions. Note that the total number of the trials in each mouse was significantly smaller in the wild-type mice (Synchronous: 89.6 ± 2.7 trials, Asynchronous: 87.5 ± 4.0 trials; mean ± standard error) than in the Caps2-KO mice (Synchronous: 103 ±3. 8 trials, *t_23_* = -2.77, *p* = 0.011 < 0.05, 95% *Confidence Interval* = [-22.8, -3.29], *d* = 1.12; Asynchronous: 104 ± 3.6 trials, *t_23_* = -3.10, *p* = 0.005 < 0.05, 95% *Confidence Interval* = [-27.5, -5.49], *d* = 1.24, two-sample t-test).
